# Perception of Musical Tension in Cochlear Implant Listeners

**DOI:** 10.3389/fnins.2019.00987

**Published:** 2019-09-20

**Authors:** Steffen Spangmose, Jens Hjortkjær, Jeremy Marozeau

**Affiliations:** Hearing Systems Group, Department of Health Technology, Technical University of Denmark, Lyngby, Denmark

**Keywords:** cochlear implant, music perception, musical tension, hearing impairment, music enjoyment

## Abstract

Despite the difficulties experienced by cochlear implant (CI) users in perceiving pitch and harmony, it is not uncommon to see CI users listening to music, or even playing an instrument. Listening to music is a complex process that relies not only on low-level percepts, such as pitch or timbre, but also on emotional reactions or the ability to perceive musical sequences as patterns of tension and release. CI users engaged in musical activities might experience some of these higher-level musical features. The goal of this study is to evaluate CI users' ability to perceive musical tension. Nine CI listeners (CIL) and nine normal-hearing listeners (NHL) were asked to rate musical tension on a continuous visual analog slider during music listening. The subjects listened to a 4 min recording of Mozart's Piano Sonata No. 4 (K282) performed by an experienced pianist. In addition to the original piece, four modified versions were also tested to identify which features might influence the responses to the music in the two groups. In each version, one musical feature of the piece was altered: tone pitch, intensity, rhythm, or tempo. Surprisingly, CIL and NHL rated overall musical tension in a very similar way in the original piece. However, the results from the different modifications revealed that while NHL ratings were strongly affected by music with random pitch tones (but preserved intensity and timing information), CIL ratings were not. Rating judgments of both groups were similarly affected by modifications of rhythm and tempo. Our study indicates that CI users can understand higher-level musical aspects as indexed by musical tension ratings. The results suggest that although most CI users have difficulties perceiving pitch, additional music cues, such as tempo and dynamics might contribute positively to their experience of music.

## Introduction

The perception and enjoyment of music can be challenging for hearing-impaired listeners. Apart from reducing sensitivity, hearing impairment can distort perceptual features important for music, including abnormal perception of loudness (Marozeau and Florentine, 2007), pitch (Moore and Carlyon, 2005) and timbre (Emiroglu and Kollmeier, 2008). Furthermore, hearing devices, such as hearing aids or cochlear implants (CI) typically alter the signal to improve speech perception rather than focusing on musical features (Fitz and McKinney, 2010; Marozeau et al., 2014). Specifically, studies have shown that CI users have difficulty perceiving pitch, identifying musical instruments, or segregating simultaneous melodies (for a review see McDermott, 2011). However, despite those limitations, it is not uncommon to find CI users engaged in musical activities, either listening at home, attending a concert, or actively playing an instrument (Gfeller et al., 2000; Migirov et al., 2009). Given many CI users' engagement with musical activities, it is worthwhile investigating the musical features that might contribute to CI users' music perception. In this study, specifically, we will investigate how CI listeners (CIL) perceive musical tension.

The CI is a medical device dedicated to restoring speech perception in people with severe hearing impairment. It is composed of a sound processor, worn behind the ear, and a receiver located between the skin and the temporal bone. The receiver is connected to an array of 12–22 electrodes inserted in the scala tympani of the cochlea. Each electrode activates different regions of the auditory nerve. As the cochlea is organized tonotopically, the sound induced by an electrode should decrease in pitch as the electrode is inserted further into the cochlea. However, as those electrodes are limited in number and are not in direct contact with individual auditory nerve fibers, they cannot restore the frequency resolution needed to convey complex pitch information. Consequently, CI users have poor pitch discrimination, and most CI users cannot identify the direction of a pitch change for steps smaller than half an octave (Looi et al., 2004). This also prevents them from correctly identifying well-known melodies without additional lyrics and rhythm cues (Gfeller et al., 2002). Furthermore, CI users have greatly impaired perception of dissonance and consonance in chord changes (Caldwell et al., 2016) as well as impaired perception of timbre (Marozeau and Lamping, 2019). However, the CI device conveys precise temporal information. Studies using rhythm discrimination tasks show similar performance scores for CI and NH listeners (Kong et al., 2004).

Listening to music is an experience that arises from more than the sum of the sensations induced by its fundamental elements: pitch, timbre, rhythm. Listening to music is a pleasant experience that can evoke emotions or memories and can bring the listener to a specific mental state. If some CIL are still engaged in musical activities, it is likely because some of these more complex aspects of musical experiences are preserved. CI users can benefit from sung lyrics in music, allowing them to enjoy the content of the songs despite being unable to identify the melody. For music with a specific rhythm or groove, CIL might enjoy the rhythm and may, for instance, become motivated to dance (Au et al., 2012). They may also use tempo and rhythm cues to evaluate the emotional mood of the song (Vannson et al., 2015). However, it is still unknown to what extent they experience the complex cognitive structure that music enjoyment also relies on.

The experience of meaningful musical structure is often described as the experience of moments of tension and release (Schenker, 1935; Schoenberg, 1975; Lerdahl, 2001). Musical tension can be created by subtle musical cues that break the listener anticipation, for example, a dissonant chord or a delayed resolution. In tonal harmony, the resolution of a dominant chord on the tonic, for instance, is typically described as a release of tension. More formal descriptions of tension have been based on tonal music theory (Lerdahl and Jackendoff, 1983; Lerdahl, 2001; Lerdahl and Krumhansl, 2007) or psychological accounts of expectation (Narmour, 1990; Margulis, 2005). Hjortkjær (2011) proposed a parametric model that predicts continuous tension ratings of NHL from musical audio features. In this model, tension is predicted as a combination of low-level acoustic features including measures of intensity variation and distribution of spectral energy, as well as higher-level features related to changes in pitch class distribution and in tonality. Of these cues, CIL might be able to perceive intensity variations and coarse spectral changes (McKay, 2004) but are unlikely to have access to higher-level features that rely on accurate pitch processing. It is thus possible that CIL can use low-level cues related to loudness and spectral variation to judge musical tension. On the other hand, it is unlikely that they will have access to cues associated with the complex tonal structure of musical pieces that are thought to be central to the perception of musical tension in normal-hearing listeners.

The perception of musical tension is often linked to musical affect (Krumhansl, 1996), but tension does not necessarily directly predict musical preference or enjoyment. In a recent study, Vannson et al. (2015) asked CIL to rate their musical preferences on 24 different, unfamiliar piano pieces. The results were strongly correlated with the regularity of tempo and rhythm. CIL reported that they enjoyed musical compositions with faster tempi and more complex rhythms more than pieces with slower tempi and regular rhythms. Such results differed from the rating of NHL that enjoyed both fast and slow pieces.

In this study, we investigated musical tension and enjoyment ratings in CIL and normal-hearing listeners, NHL, controls. We examined the effects of selectively manipulating cues related to intensity, pitch, rhythm, and tempo that are each important for tension and enjoyment in NHL. We hypothesized that CIL would rely mostly on loudness and temporal cues in their tension ratings and that more regular rhythms would reduce the overall enjoyment of the music.

## Methods

### Participants

Eighteen volunteers, divided into two groups, participated in this study. The first group consisted of 9 NHL (six males, three females, age range 23–31, mean age, 27) with audiometric threshold levels between −10 and 20 dB HL at octaves from 250 Hz to 8 kHz. The second group consisted of three bimodal and six bilateral CI recipients (six females, three males, age range 23–79, mean age 47.9). Seven of the CI participants were post-lingually deaf and 2 were pre-lingually deaf, all with more than 1 year of experience with the implant. They were all fitted with Cochlear Ltd devices (Freedom Contour Advance or newer, equipped with a CP800 or newer, fitted with ACE) except for one Medel user (Flex 24 with CP910) and one with Advanced Bionics (HiRes 90K with Naida CIQ70). One of the three bimodal users was fitted with a Phonak Naída hearing aid. All of the participants had none or <3 years of musical training. They provided informed consent before the study, and all experiments were approved by the Science-Ethics Committee for the Capital Region of Denmark (reference H-16036391).

### Stimuli

The stimuli were based on W. A. Mozart's *Piano Sonata, No. 4 E*♭ *major, K282*. This piece has been used in several previous studies on musical tension in NHL wherein elaborate analyses of the musical structure exist (Krumhansl, 1996; Lerdahl, 1996, 2001; Narmour, 1996; Vega, 2003; Margulis, 2005; Hjortkjær, 2011). The piece was recorded by a trained pianist on an acoustic piano with 88-weighted keys (Roland V-piano), equipped with MIDI sensors that allow storing of all MIDI information: note-on event, note-off event, velocity, and foot controller activation. Audio files of the stimuli can be downloaded from the Supplementary Material section. Five stimuli were constructed: the baseline (original piece) and four modified versions: random notes melodies, fixed intensity, fixed tone duration, and increasing tempo changes. All versions were rendered based on the MIDI information using samples of a Steinway Grand Piano. In the four modified versions, specific musical features were altered on the MIDI information before the resynthesis. All the stimuli were 190 s in duration except condition 4, which had a duration of 275 s. All stimuli had a dynamic range of 30 dB except for condition 3 (fixed intensity), which had a dynamic range of 19 dB.

The four different stimulus manipulations were designed to examine the relative influence of various musical features on the tension ratings, including tonality, intensity, rhythm, and tempo. In the first manipulation, the pitch of each tone was randomized based on a uniform distribution within the melodic range of the original piece (*Eb*_1_ to *Bb*_5_). This manipulation removed both melody and tonal cues while preserving the original rhythm and intensity cues. In the second manipulation, each note onset was set to have equal intensity (MIDI velocity value 77 corresponding to mid-range intensity). Small local differences in perceived loudness could still persist due to potential loudness summation between simultaneous and overlapping notes and the dependency of loudness on frequency. Temporal irregularities of the baseline were removed by quantizing each note to the closest 1/16 note. In the third manipulation, the duration between tones was set to a fixed duration equivalent to a half-note (0.78 s at 154 bpm), and rest intervals were set to a constant of a 3/16 note (0.29 s). This manipulation disrupts temporal information and keeps the density of note events at a fixed level. Tone pitches were unaltered in this condition, but the manipulation of note onsets naturally also alters the perceived melody. The fourth and last manipulation artificially enhanced tempo variations relative to the original performed piece. Using the MIDI tempo track, the tempo was scaled so that the tempi of faster sections were further increased, and slower sections further decreased. This modification, therefore, exaggerated tempo changes.

### Procedure

Participants were seated in a soundproof booth 1.5 m from a nearfield loudspeaker (Dynaudio Acoustics BM6) and presented with the different stimuli in random order. For each stimulus, participants were instructed to continuously rate the amount of perceived tension throughout the piece by adjusting the position of a vertical physical slider (Evolution MIDI controller, UC-33) placed in front of them. No explicit definition of musical tension was given to the participants to be consistent with previous studies and to avoid biases (Madsen and Fredrickson, 1993; Krumhansl, 1996; Hjortkjær, 2011). Previous work found that the instruction to rate tension yielded ratings that are consistent across listeners (Fredrickson, 1995; Lychner, 1998), and that more explicit definitions could bias the results (Hjortkjær, 2011). The slider was positioned at the lower extreme (low tension) at the onset of each piece. The experiment consisted of three blocks each comprising all the stimulus conditions presented in random order. So each stimulus was presented three times in total. After each stimulus, participants were told to rate the piece using another continuous vertical slider ranging from: “do not like at all” to “like very much.” Overall the experiment lasted ~1 h, including a small break between blocks.

Bilateral CIL were instructed to turn off their processor on their least favorite hearing site. Bimodal CIL were fitted with an earplug to minimize the contribution of any residual hearing. All NHL were equipped with an ear-plug in their left ear. The CIL were told to use their preferred setting for music listening.

The experiment interface was designed in Pure Data (version 0.47.1), and the position of the slider was recorded every 200 ms with 7-bits precision integers.

The stimuli gain were set such that the L_Aeq,peak_ through the total duration of the piece did not exceed 65 dB SPL measured with a sound level meter (B&K type 2250), repeated for all conditions. This level was selected because sounds at 65 dB SPL are mapped by default to the C-level (maximum possible current) of Cochlear Ltd devices.

Participants received no information about the different structure of the five different stimuli prior to the experiment.

### Analysis

To compare continuous tension ratings across conditions that included different tempi, the rating values were interpolated per beat. In total, 128 sample values were used in the analysis corresponding to 32 measures with a 4/4 metric.

Non-parametric permutation tests were used to identify statistical differences in the tension ratings between conditions or between groups. For each time sample in the tension ratings, we randomly permuted the group or condition labels 100,000 times to generate a null distribution of group/condition differences. Differences in the tension ratings exceeding *p* < 0.05 of the null distribution were considered significant.

The correlation between tension ratings between groups was assessed with permutation-based statistics. To compare tension rating curves of the two groups, we first generated phase-scrambled versions of the ratings by randomizing the phase in the Fourier domain. This creates random data with the same frequency content as the original ratings. To generate a null distribution, we computed Pearson correlation coefficients between the true and phase-scrambled rating data 10,000 times. A *p*-value for the true correlation was then calculated as (b + 1)/(m + 1) where *b* is the number of random correlation values that exceeds the true correlation, and *m* is the total number of random permutations (Phipson and Smyth, 2010). This approach generates an estimate of the strength of the correlation that is unbiased by the auto-correlated nature of the tension ratings.

Enjoyment ratings were compared with Student *T*-tests. Within each group, the baseline was compared with the other four conditions using a two-tail paired Student *T*-test. Differences across groups in the baseline condition were compared with a two-sample Student *T*-test. To minimize the risk of *False Positive* due to multiple comparisons, *p*-values below 0.01 were considered significant (0.05 divided by the number of tests). Due to a technical problem, the enjoyment rating scores were saved for only 7 out of 9 CIL.

## Results

### Condition 1: Baseline—The Unaltered Piece

In this condition, listeners rated the original piece without modifications. The piece was rendered from the recorded MIDI that includes information about note-on and -off events, the key signatures, velocity, and foot controller.

Consistency within groups of listeners was tested using a hierarchical cluster tree analysis. The analysis revealed that the ratings of one of the CI participants (CI4) was highly uncorrelated with the remaining CI participants. Furthermore, this listener also showed larger variability of rating across repetition of the same condition. Unlike the remaining participants, this listener had considerable difficulties understanding the task during the test. For these reasons, this listener was considered an outlier and was excluded from the rest of the analysis. The results from the other listeners were averaged within their groups and across repetitions.

**Figure 1:**
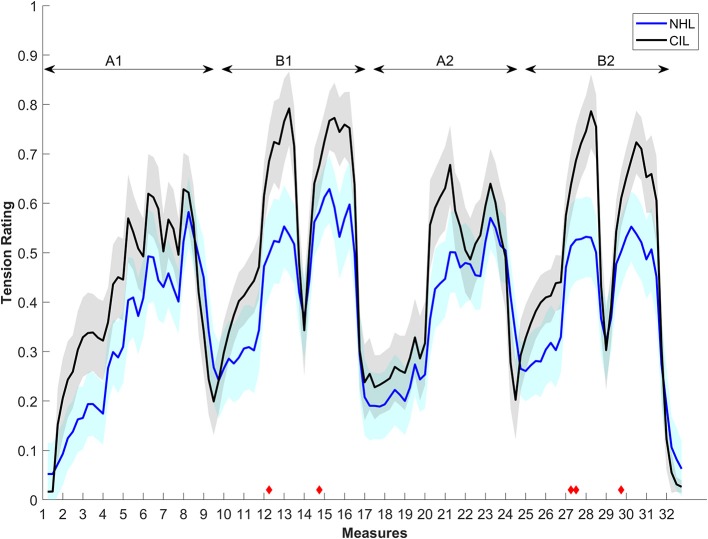
Average tension ratings of 8 CIL (in black) and 9 NHL (in blue) as a function of the musical measures of the non-modified piece (baseline). Shaded areas represent standard errors. Red diamonds indicate the period in which the two ratings differ significantly. Horizontal double arrows outline the 4 parts of the piece (A1, B1, A2, B2).

Figure 1 shows the average tension rating as a function of time for both the NHL and CIL. A high degree of similarity between the groups in the overall contours of the rating curves is noticeable. In both groups, the ratings qualitatively follow the build-up and release of tension within the different musical segments. The ratings between CIL and NHL were found to be strongly correlated (*r* = 0.92, *p* < 0.001). The musical piece is composed of two main sections (A and B) that are repeated. The first part of section A gradually builds up to reach a climax (measures 6, 7), and then slowly resolves (measure 9). The section B starts with low tension (measure 10), then builds a first climax (measures 12, 13) that momentary resolves (measure 14), rebuilds a second climax (measures 15, 16) and resolves shortly after (measure 17). The piece then repeats, but with slightly different interpretation by the musician. The first presentation of the sections A and B will be named A1 and B2, their repetition A2 (measures 17 to 24) and B2 (from measure 25 to 32) (further detailed musical analysis can be found in Krumhansl, 1996; Narmour, 1996; Lerdahl, 2001). Aside from the resolution of the sections A (measures 9 and 24), CI users rated the piece overall as more tense, but the ratings were generally very similar. Non-parametric permutation tests were used to assess if specific sections were statistically different. Only the climaxes in the B sections were found to be significantly different between subject groups, as indicated by the red diamonds in Figure 1.

No significant difference of the enjoyment ratings was found between the two groups of listeners [mean enjoyment ratings NHL listeners: 63.43%; CIL: 50.39%, *t*_(13)_ = 1.3835, *p* = 0.1898].

The perhaps surprisingly high degree of similarity between the ratings of CIL and NHL indicate that CIL can relate musical sequences to ebbs and flow of musical tension. However, it is unclear which cues they are using as many of the musical features that are predictive of tension ratings will covary. Therefore, the two groups could have based their judgment on different cues and still rated tension similarly. Therefore, other conditions were tested to assess the influence of different cues on the ratings.

### Condition 2: Tonal Cues—Random Note Melodies

In this condition, the listeners were presented with a modified version of the original piece, where the pitch of each tone was set to a random value within the pitch range of the original piece. All other information, such as tone onset timing and intensity variations in the performance, were kept identical to the baseline.

**Figure 2:**
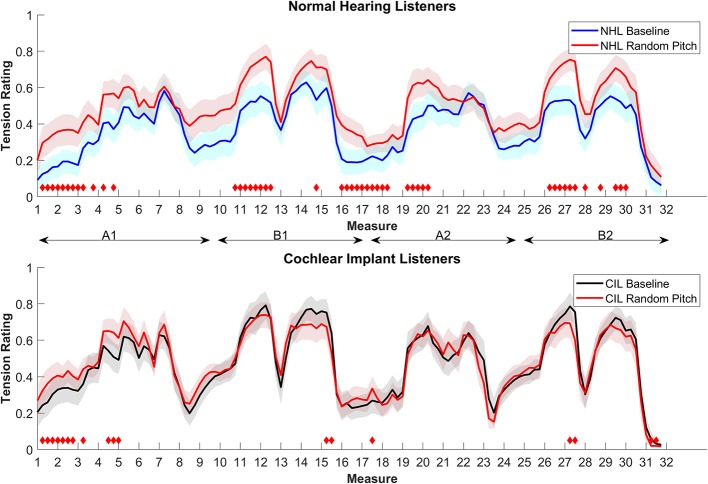
Average tension ratings of 9 NHL **(top panel)** and 8 CIL **(bottom panel)** for the baseline and condition 2, random pitch (in red). See details in Figure 1.

Figure 2 shows the results for the NHL (upper panel) and the CIL (lower panel) in comparison to their group baseline. As can be observed, removing tonal information affected the ratings of the NHL significantly but affected the ratings of CIL to a lesser degree. Overall, tension ratings were higher in the random pitch condition in the NH group, which may relate to the higher degree of overall dissonance. For the NHL, significant differences were observed between the original and random pitch stimuli throughout the piece (as indicated by the red diamonds), except for the second part of section A. On the other hand, removing tonality from the original melody had a smaller effect on CIL ratings. With the random pitch, segments were judged to be more tense than the original mostly during the first part of section A, while the climax segments in the B sections were judged to be less tense than the original. The magnitude of the root-mean-square difference in ratings between stimulus conditions was evaluated for the NHL and CIL groups; values ranged from 0 (no difference at all) to 1 (maximal possible difference). The mean difference between stimulus conditions was more than twice as large for the NHL group (0.13) than for the CIL group (0.05).

This difference in rating between CIL and NHL can also be seen within their enjoyment rating. NHL' enjoyment rating dropped significantly from 63.43% in the baseline to 28.23% [*t*_(8)_ = 3.9773, *p* = 0.0041]. On the other hand CIL' ratings stayed at a similar level at 53.28% [*t*_(5)_ = −0.9348, *p* = 0.3928].

The results of this condition support the notion that the contribution of tonal cues is different for CIL and NHL. This outcome is expected, given the weak ability of CIL to discriminate pitch and poor melody recognition. Although the tension ratings for the NHL group were upwardly shifted with the random pitch, the overall baseline contour from the original was preserved with the random pitch. This also suggests that NHL can use other cues in their tension ratings that could possibly be processed by CIL. These could relate to loudness and timing cues that were assessed in the following conditions.

### Condition 3: Loudness Cues—Fixed Intensity

The amplitude envelope of the signal can be a strong predictor of tension ratings (Hjortkjær, 2011), suggesting that intensity variation is an essential feature for musical tension. CIL should be able to perceive some form of intensity variations and may rely on them to judge musical tension. In this condition, all tones were set to the same onset velocity to remove intensity variations. Additionally, temporal irregularities of the baseline were removed by quantizing all notes at 1/16 note level. Overall, this manipulation creates artificial or “robotic” sounding stimuli, but where the tonal-rhythmic structure is preserved.

**Figure 3:**
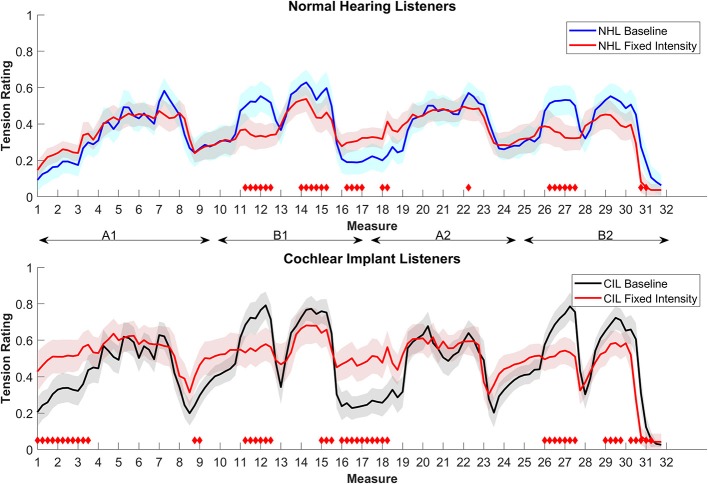
Average tension ratings of 9 NHL **(top panel)** and 8 CIL **(bottom panel)** for the baseline and condition 3, fixed intensity (in red). See details in Figure 1.

Figure 3 shows the average rating for both groups. Both groups show different ratings compared to their baselines. However, this manipulation had a more pronounced effect on the ratings of the CIL (RMS difference to the baseline for CI = 0.14, NH = 0.09). For CIL, removing intensity cues had the effect of “neutralizing” the ratings with less overall variations. This result indicates an apparent impact of loudness cues on the ratings of CIL. For NHL, removing loudness cues also had a “neutralizing” effect, but the effect was less outspoken. The larger differences for the NHL can be seen in section B in which the climax is less efficient at creating a tense sensation and at the beginning of the A2 where the release is less pronounced.

NHL' enjoyment rating dropped significantly from 63.43 to 41.99% [*t*_(8)_ = 4.1113, *p* = 0.0034]. Enjoyment ratings of the CIL also drop from 50.39 to 42.38%, although this difference was not significant [*t*_(5)_ = 0.9222, *p* = 0.3988].

### Condition 4: Temporal Cues—Fixed Tone Duration

As CIL can reliably discriminate rhythm and tempo, temporal cues might have an effect on the tension ratings. In this condition, the music stimulus was modified so that the duration of and between each note was fixed (to half-note length). This manipulation effectively removes tempo variations. Without access to tonal cues, fixed duration also removes rhythmic complexity.

**Figure 4:**
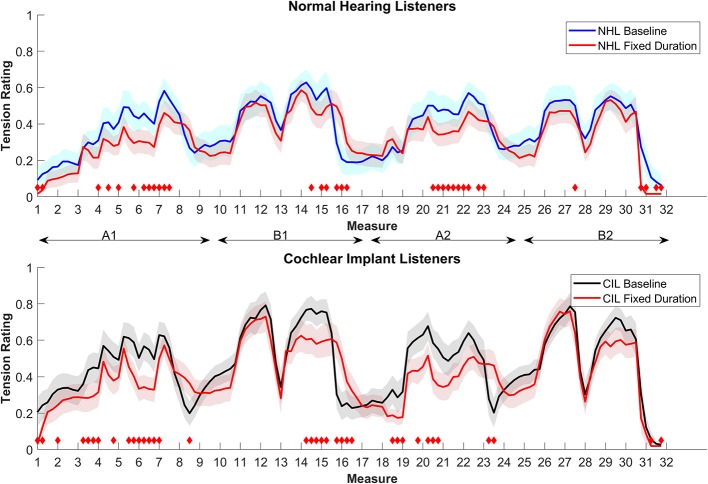
Average tension ratings of 9 NHL **(top panel)** and 8 CIL **(bottom panel)** for the baseline and condition 4, fixed duration (in red). See details in Figure 1.

Figure 4 shows the ratings for both groups. The change in temporal structure affected the ratings of both groups similarly. Mostly the climax of A and the second part of B were judged as less tense for both groups, while the transition between B1 and A2 were judged as more tense. Overall, NHL rated this condition less tense (23.62% of the time) or more tense (3.15% of the time) than baseline (RMS difference = 0.08). CIL judged this condition 22.05% of the time as less tense, and 5.51% of the time as more tense (rms of the difference 0.11). It is interesting to notice that the change in temporal cues had a similar effect on both groups, given that it has been showed that CIL have similar abilities as NHL in rhythm discrimination (Kong et al., 2004).

The enjoyment ratings were not significantly changed relative to the baseline for either group.

### Condition 5: Increased Tempo Changes

**Figure 5:**
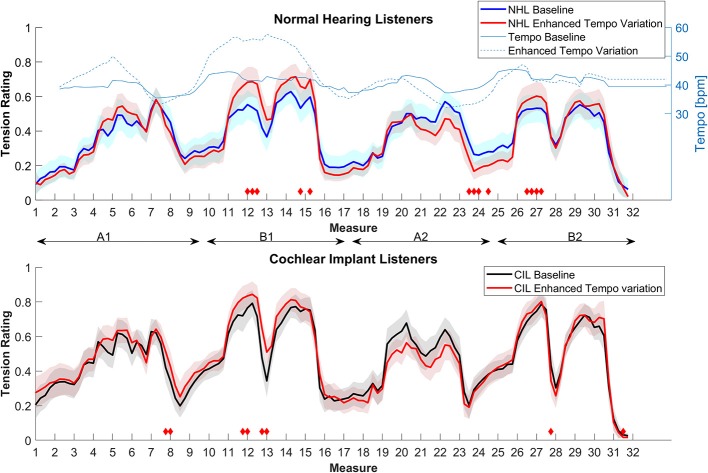
Average tension ratings of 9 NHL **(top panel)** and 8 CIL **(bottom panel)** for the baseline and condition 5, enhanced tempo variation (in red). See details in Figure 1.

This last condition was designed to test whether tension ratings can be influenced by tempo changes. Many styles of music allow for the tempo to vary quite dramatically, which can be used to convey tension or emotional content. In this condition, tempo variations of the baseline were artificially enhanced. The parts in which the performer slowed down were further reduced in tempo, and vice-versa. All other musical information was kept similar. Figure 5 shows the tempo map of this condition in comparison to the baseline.

Figure 5 shows the average ratings for both groups. Overall, the differences in ratings were relatively small for both groups compared to their respective baselines. Nevertheless, some significant differences were observed. For the NHL, the modified tempo had the expected effect. A substantial increase of tempo during the section B1 induced a significant rise in tension (overall 7.09% more tense) and decreases in tempo between the measure 20 to 26 caused a significant lower tension rating (overall 3.15% less tense). The enhanced tempo variation also affected the ratings of the CIL but less so compared to the NHL (overall 4.72% more tense, and 1.57% less tense).

Enjoyment scores did not differ from the baseline for either group.

## Discussion

In this study, we compared continuous ratings of musical tension in CIL and NH controls to better understand what dimensions of musical experience might be preserved in CIL. In the unaltered baseline, the musical tension ratings of CIL were strikingly similar to those of NHL. The essential shape of the tension curves that define long-term ebbs and flow of tension throughout the musical piece was qualitatively similar in the two groups (Figure 1). This clearly suggests that CIL experience different levels of musical tension. This result is perhaps surprising, and is not, to the authors' knowledge, previously reported in the literature. Given the difficulty of CIL to perceive pitch and harmony, it would not have been surprising to find that CIL rate tension very differently from NHL. It worth noting that the notion of musical tension was not explicitly defined in any particular way to avoid biasing the participants. Therefore, it is possible that CIL and NHL interpreted the concept of tension differently despite similar ratings.

**Figure 6:**
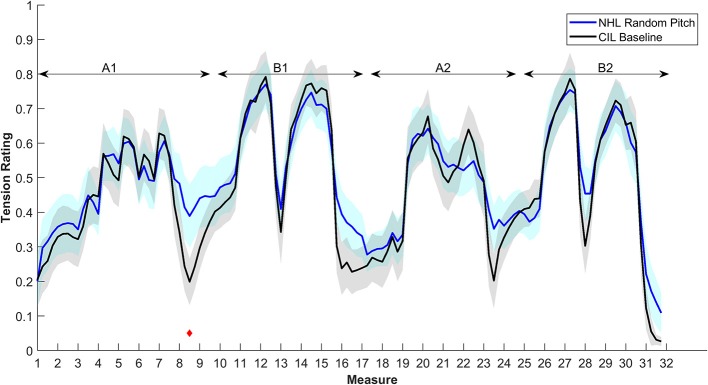
Average tension ratings of 9 NHL for condition 2, random pitch (in blue), and 8 CIL for the baseline (in black). See details in Figure 1.

Although the average pattern of responses is very well-correlated with the one from NHL, Figure 1 also shows that CIL rate the piece as more tense overall. This could potentially relate to their perception of the individual piano tones. It is not straightforward to evaluate how CIL perceive piano tones. However, a study on CI users with residual hearing suggested that they perceive a pulse train presented on a single electrode as an inharmonic sound (Lazard et al., 2012). It is, therefore, reasonable to assume that CIL will perceive single piano notes as inharmonic sounds as well. An overall more dissonant perception could potentially explain the overall increased tension ratings. In this case, the Random pitch condition should produce very similar patterns since NHL also lack tonal cues in that condition. Figure 6 shows that the magnitudes of tension rated by the CIL during the baseline is indeed very similar to that of the NHL in the Random pitch condition, except for the measures linked to a release of tension after a climax (measures 8, 13, 16, 23, and 27).

**Figure 7:**
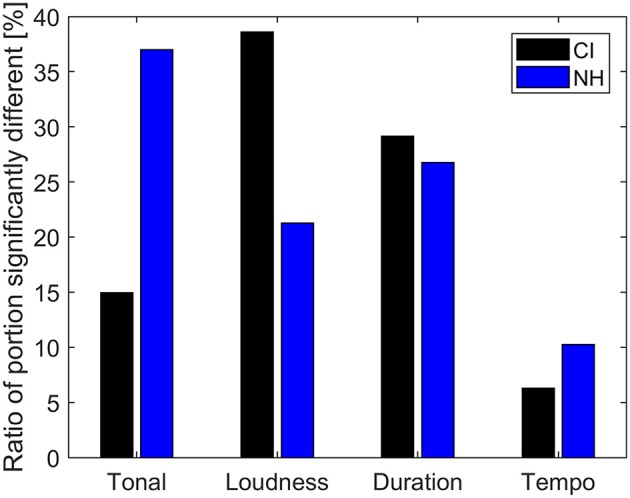
Ratio of rating significantly different from the baseline for the four modified conditions: random pitch (Tonal), fixed intensity (Loudness), fixed duration (Duration) and enhanced tempo variation (Tempo).

We hypothesized that CIL would rely mostly on loudness variations and not on features that depend on the pitch in their tension ratings. The ratings of the musical stimulus with loudness variations reduced (Figure 3) support this showing a large difference from the ratings of the unaltered baseline piece. However, Figure 2 shows that the ratings were also affected by the change in tonal cues. Figure 7 summarizes the effect of the different conditions by showing the percentage of significant change for each condition. This indicates that removing loudness cues was the manipulation that had the largest impact on the tension ratings for the CIL and more than twice the effect of pitch cues. The trend is the opposite for the NHL where ratings were strongly affected by the removal of pitch cues and less so by removing loudness cues. The results indeed suggest that the perception of musical tension is dominated by loudness variations for the CIL, but also suggest a small impact of pitch variations. However, the effect of randomizing the pitch also affects the perception of overall loudness in the CIL. In the Cochlear device, the cut-off frequency of the lowest filter band is set by default to 188 Hz, which roughly correspond to *F*_#3_. Therefore, only the harmonics of the notes below that limit will be transmitted. Given that in condition 3, the notes were randomly assigned to a value between *Eb*_1_ to *Bb*_5_, it can be assumed that the loudness of many notes was altered.

Additionally, the small but significant effect of pitch that was observed in CIL might have been caused by the method that was used to randomize the notes. To generate random sequences, each note was picked randomly based on a uniform distribution within the original melodic range from *Eb*_1_ to *Bb*_5_. This introduces larger step intervals between consecutive notes compared to the distribution of step intervals in the original piece (and in tonal music in general). This might have introduced pitch changes that are more salient when perceived through the cochlear implant, which could have influenced the ratings of the CIL. It would have been possible instead to generate random sequences with a distribution of smaller step sizes which would still have destroyed the tonal cues. We would thus expect CIL ratings of random melodies with smaller pitch changes to be more similar to the baseline condition as CIL might not have perceived differences in pitch at all.

The sound level was set to optimize the perception of the dynamic range of the musical stimulus. We assigned a maximum level to 65 dB SPL since levels exceeding 65 dB are heavily compressed by the Cochlear device. At the other end, sounds at a level below 25 dB SPL are not transmitted. An acoustic analysis of the baseline stimulus showed that the dynamic range of the piece was 30 dB, meaning that a maximum level of 65 dBA ensures that the soft parts can be clearly perceived. Since CIL appear to rely heavily on loudness cues, the level of presentation is likely to be very important. This study underlines the importance of presenting the music at an optimal level to optimize the perception of the musical dynamics. Additionally, it also suggests that compression stages should be considered with care, as they might alter this important cue.

Previous work by Vannson et al. (2015) indicated that CIL preferred music with faster tempi and more complex rhythm. Based on this, we investigated whether exaggerating tempo changes would enhance tension and enjoyment ratings in the CIL and whether removing rhythmic cues and tempo variations reduce them. However, removing or exaggerating tempo variations had only little effect on the ratings in either group. Tension ratings decreased in a similar way for NHL and CIL (Figure 4) in the fixed duration condition. However, artificially increasing tempo variations had disappointingly minor effects on either tension or enjoyment ratings, which were not significantly different from the baseline condition.

**Figure 8:**
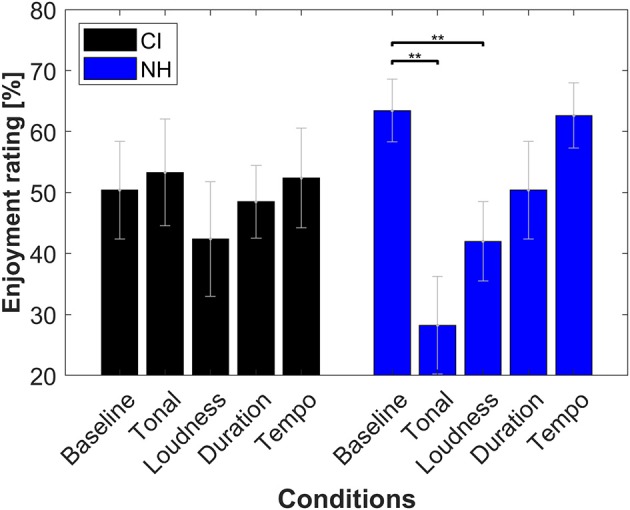
Average enjoyment ratings for each condition of 6 CIL (in black) and 9 NHL (in blue). Horizontal lines and asterisks indicate significant differences (*p* < 0.01). See details in Figure 7.

**Figure 9:**
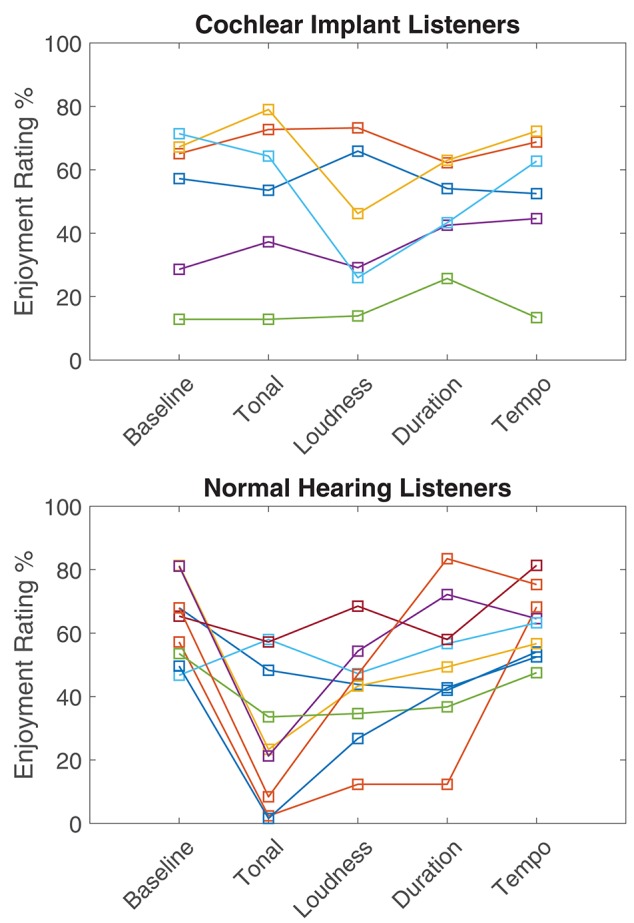
Individual enjoyment ratings for each participant (represented by different colors). See details in Figure 7.

Overall, the different conditions had little effect on the enjoyment rating for CIL. Figure 8 summarizes the average rating for each condition and group of listeners. The only statistical difference found was between the baseline and conditions 2 and 3 (tonal and intensity cues) for the NH group. However, a large variability can be observed in both groups. A large spread of music enjoyment ratings in CIL has already been reported in the literature (for example, see Migirov et al., 2009). Figure 9 shows the individual enjoyment rating for each condition and group. Inside the CI group, the enjoyment rating of the baseline ranges from 12 to 71%. The different conditions did not affect those ratings except for two CIL that showed a large decrease of enjoyment for condition 3 (Loudness fixed). On the other hand, condition 2 (Tonal cues) was rated as dramatically less enjoyable for half of the NHL, while remaining stable for the other half. It is possible that this difference in ratings could be related to the listeners' familiarity with contemporary music.

If a musical piece is viewed as a mixture of the structure written by the composer and the interpretation of the musician, condition 3 (fixed intensity) can be seen as keeping only the effect of the composer (same tones and rhythm) and removing the effect of the musician. On the other hand, condition 2 (random notes) can be seen as keeping only the effect of the musician, while stripping away the work of the composer. This study shows that NHL rely on both aspects to enjoy and the experience music. Since the performance follows the dynamical structure of the composed music, features, such as loudness and tempo variations also convey the tension and release patterns of the music. CIL appear to be able to perceive the tension-release structure via such cues even without access to the tonal structure of the music. This might explain why CIL often enjoy music listening despite their poor perception of pitch.

## Conclusions

Despite a poor perception of pitch and harmony, many cochlear implant, CI, users enjoy music. In this study, we show that CI users rate musical tension in a very similar way as normal-hearing listeners. By modifying the music on different dimensions, our results indicate that CI users rely mostly on rhythm and intensity cues to judge musical tension. This suggests that the perception of music can be conveyed by other features than the tone pitch itself. The importance of intensity variations for CI listeners also underlines the importance of listening to music at an appropriate level and without excessive compression.

## Data Availability

The datasets generated for this study are available on request to the corresponding author.

## Ethics Statement

The studies involving human participants were reviewed and approved by Science-Ethics Committee for the Capital Region of Denmark (reference H-16036391). The patients/participants provided their written informed consent to participate in this study.

## Author Contributions

JM initiated the project. SS designed the experimental interface, recruited the participant, and collected the data. This experiment was performed during SS master's final research project supervised by JM. All the authors have contributed toward the experimental design, the analysis, and the writing of the paper.

### Conflict of Interest Statement

The authors declare that the research was conducted in the absence of any commercial or financial relationships that could be construed as a potential conflict of interest.
